# Immunological correlates of behavioral problems in school-aged children living with HIV in Kayunga, Uganda

**DOI:** 10.1017/gmh.2015.7

**Published:** 2015-06-25

**Authors:** H. Ruiseñor-Escudero, I. Familiar, N. Nakasujja, P. Bangirana, R. Opoka, B. Giordani, M. Boivin

**Affiliations:** 1 Department of Psychiatry, East Lansing, Michigan State University, Michigan, USA; 2 Department of Psychiatry, Makerere University, Kampala, Uganda; 3 Department of Psychiatry, University of Michigan, Michigan, USA

**Keywords:** HIV, CBCL, BRIEF, neurodevelopment, Uganda

## Abstract

**Background.:**

HIV can affect the neuropsychological function of children, including their behavior. We aim to identify immunological correlates of behavioral problems among children living with HIV in Uganda.

**Methods.:**

Children participating in a parent randomized control trial in Kayunga, Uganda were assessed with the Behavior Rating Inventory of Executive Function (BRIEF) and the Child Behavior Checklist (CBCL). We constructed simple and multiple linear regression models to identify immunological correlates of behavioral problems.

**Results.:**

A total of 144 children living with HIV (50% male) with a mean age of 8.9 years [Standard Deviation (s.d.) = 1.9] were included in the analysis. Eighty-two children were on antiretroviral therapy. Mean CD4 cell count % was 35.1 cells/μl (s.d. = 15.0), mean CD4 cell activation 5.7% (s.d. = 5.1), mean CD8 cell activation was 17.5% (s.d. = 11.2) and 60 children (41.7%) had a viral load of <4000 copies/ml. In the adjusted models for the BRIEF, higher scores were associated with higher viral loads (aβ = 16.7 × 10^−6^, 95% CI −5.00 × 10^−6^ to 28.4 × 10^−6^), specifically on the behavioral regulation index. Higher mean CD8 activation % was associated with higher scores on the Externalizing Problems  and Total Problems  scales of the CBCL (aβ = 0.17, 95% CI 0.04–0.31 and aβ = 0.15, 95% CI 0.00–0.28, respectively).

**Conclusions.:**

Poorer behavioral outcomes were associated with higher viral loads while higher CD8 activation was associated with poorer emotional and behavioral outcomes. Complete immunological assessments for children living with HIV could include commonly used viral and immunological parameters to identify those at higher risk of having negative behavior outcomes and who would benefit the most from behavioral interventions.

## Introduction

According to Joint United Nations Program on HIV/AIDS (UNAIDS) there were 190 000 children (170 000–220 000) 0–14 years of age living with HIV in Uganda (Joint United Nations Program on HIV/AIDS, 2015). As a result of the national scale-up of antiretroviral therapy (ART), the number of children living with HIV is increasing (UNAIDS, 2013), which highlights the importance of being able to identify those at higher risk of developing complications associated with HIV infection, such as neurobehavioral deficits (Laughton *et al*. 2013).

HIV is a neurotropic virus that invades the central nervous system (CNS) early after infection, primarily via infected monocytes/macrophages and CD4^+^ lymphocytes (Kovacs 2009). The psychological effects of HIV on children are frequent, ranging from mild to severe (Mellins *et al*. 2003; Wolters & Brouwers, 2005; Nachman *et al*. 2012). Developmental deficits among HIV-1 infected children include: language and motor skills (Ultmann *et al*. 1987; Drotar *et al*. 1997), verbal and memory deficits (Levenson *et al*. 1992), visual spatial integrative ability (Boivin *et al*. 1995; Laughton *et al*. 2013) and executive functions (Bisiacchi *et al*. 2000). In addition, they are at higher risk of psychiatric and mental health problems (Laughton *et al*. 2013), delinquent behavior and poor social competence (Bomba *et al*. 2010). Although dementia and encephalopathy are now rare in developing countries with the introduction of antiretrovirals (ART) (Ghafouri *et al*. 2006), cognitive problems, developmental delays, behavioral, psychiatric and motor problems are still common among children living with HIV who have been infected perinatally. Research suggests that this might be related to host-related factors, including T-cell activation (Jennings *et al*. 1994). However, other biological and environmental causes should also be considered as potential risk factors for poor behavioral outcomes (Mellins & Malee, 2013; Llorente *et al*. 2014).

HIV biomarkers (e.g. viral load and HIV-1 subtype) and immunological parameters (e.g. CD4^+^ and CD8^+^ T-cell count) have been identified as independent markers of disease progression (Sanchez-Ramon *et al*. 2003; Sacktor *et al*. 2009; Boivin *et al*. 2010; Tobin & Aldrovandi, 2013). Higher HIV viral loads have been associated to poor neuropsychological function among children and adolescents (Jeremy *et al*. 2005), while CD4^+^ and CD8^+^ T-cells are fundamental for HIV infection control, with their efficiency fluctuating from time of infection and specific type of surface receptors (Appay & Sauce, 2008). Higher CD4^+^ and CD8^+^ T- cell counts have been associated with better neurobehavioral functioning (Marcotte *et al*. 2003; Sanchez-Ramon *et al*. 2003). In general, CD8^+^ CD38^+^ HLADR^+^ subsets, which are markers of activated CD8^+^ T-cells, are observed in more advanced disease stages (McCloskey *et al*. 2001). Kapetanovic *et al*. reported in 2012 that higher CD4^+^ CD38^+^ HLADR^+^ T-cells had a neuroprotective effect in HIV infected children younger than 1 year of age (Kapetanovic *et al*. 2012). Mekmullica *et al*. reported a favorable association between low immune activation and the psychomotor developmental index of the Bayley scales of infant development through the third year of life, as measured by ≤5% CD8^+^HLA-DR^+^ among HIV infected children <2 months (Mekmullica *et al*. 2009).

Recent research provides evidence of behavioral problems associated with attention among HIV infected children (Mellins *et al*. 2012). Additionally, compromised executive function and slowed information processing have also been reported (Wachsler-Felder & Golden, 2002). Such findings underscore the importance of examining behavior, in addition to cognition in children living with HIV. However, it is important to highlight that research aimed at identifying risk factors for behavioral problems among this population has shown mixed results and are limited in their comparability (Mellins & Malee, 2013).

Several tools have been used to measure behavioral problems in children living with HIV (Black *et al*. 2007) some of them tested and validated for their use in sub-Saharan Africa (Bomba *et al*. 2010; Tadesse *et al*. 2012; Boivin *et al*. 2013). The Behavior Rating Inventory of Executive Function (BRIEF) (Gioia *et al*. 2000) and the Achenbach Child Behavior Checklist (CBCL) (Achenbach, 1991) are among the most widely used measures to assess behavioral, emotional, social and functional problems in school aged children. The BRIEF was designed to screen for emotional and behavioral aspects of a child's executive functioning as assessed from the perspective of the principal caregiver or parent. In contrast, the CBCL was designed to more broadly screen for emotional and behavioral psychiatric symptoms in children.

Given the important behavioral problems identified among HIV infected children and the clinical relevance associated to CNS involvement, our objective was to identify viral and immunological biomarkers of HIV disease associated with poor neurodevelopmental outcomes among school-aged children living in Kayunga, Uganda, as measured by the BRIEF score and the CBCL.

## Methods

### Study design

This is a secondary data analysis of baseline data collected from children aged 5–12 years participating in a randomized-controlled trial exploring the effects of a Computerized Cognitive Rehabilitation Training (CCRT) program for HIV infected children aiming to evaluate the effectiveness of CCRT in improving the cognitive performance outcomes and reducing the psychiatric symptoms among Ugandan children living with HIV. Participants were screened and recruited in Kayunga district (80 km northeast of Kampala) between 2010 and 2013. Children with a medical history of serious birth complications, severe malnutrition, bacterial meningitis, encephalitis, cerebral malaria, or other known brain injuries or disorders requiring hospitalization were excluded. A total of 144 HIV infected children were enrolled at baseline. All children were perinatally-infected and confirmed as HIV-positive with a Western Blot or ELISA test.

Our aim was to identify the viral and immunological biomarkers of HIV disease associated with poor neurodevelopmental outcomes among school-aged children living in Kayunga, Uganda as measured by the BRIEF score and the CBCL.

### Study procedures

Written consent was obtained from the parent/guardian and assent from children 7 years and older. After administering the informed consent, child testing and caregiver questionnaires were done in Luganda, the local language spoken in Kayunga district, in a private and quiet setting inside the project's office. Peripheral blood draws were performed in all children to determine complete blood cell count and immunologic biomarkers, including CD4^+^ and CD8^+^ T- cell counts and viral load. Blood specimens taken at Kayunga District Hospital were transported to the Makerere University-Walter Reed Project (MU-WRP).

The Institutional Review Boards of Michigan State University, University of Michigan, the School of Medicine Research Ethics Committee at Makerere University, and the Ugandan National Council for Science and Technology approved the study.

### Viral load assessment

Viral load was performed using the Amplicor HIV-1 Monitor test, version 1.5 (Roche Diagnostics, Indianapolis, IN) in the standard mode.

### Assessment of CD4^+^ and CD8^+^ T-cell activation

Imunophenotyping of CD4^+^ and CD8^+^ T-cells was performed. Samples were processed at MU-WRP. The current analysis focuses on two phenotypes: CD4^+^ CD38^+^ HLADR^+^ and CD8^+^ CD38^+^ HLADR^+^.

To measure immune activation, peripheral blood mononuclear cells (PBMCs) were isolated by ficoll-hypaque gradient centrifugation. Freshly isolated PBMCs were analyzed for CD4^+^ and CD8^+^ T cell activation using the Beckton-Dickinson FACScalibur (BD, San Carlos, California). Cells were stained with fluorochrome monoclonal antibodies: CD4^+^ or CD8^+^ PerCP-cy5.5, HLA-DR PE and CD38 APC. Preset gating was applied to all samples based on a naturally occurring break in the expression pattern of the activation markers documented in healthy Ugandan children.

### Behavioral assessments

The CBCL for school-age children (6–18 years) was administered to the principal caregiver. It is a paper-pencil parent/caregiver report on child behavior consisting of 120 items scores on a three-point Likert scale (0 = absent, 1 = occurs sometimes, 2 = occurs often). The time frame for the item responses is the past 6 months. The instrument is organized in 8 syndrome scales (Anxious/Depressed, Depressed, Somatic Complaints, Social Problems, Attention Problems, Thought Problems, Rule-Breaking Behavior, Aggressive Behavior) that group into higher order factors-internalizing (emotional problems) and Externalizing Problems [EP (behavioral problems)], respectively, and into a summary score; Total Problems [TP (internalizing plus externalizing, plus sleep and other Problems)]. It has been widely used as rating scale in different context including Ugandan children, where test-retest and internal reliability were 0.64 and 0.83, respectively (Bangirana *et al*. 2009*a*, *b*). The CBCL was translated into Luganda by a research assistant and then back-translated to English by a different research assistant, both fluent in Luganda and English. A psychiatrist fluent in both Luganda and English compared the two English versions and resolved any discrepancies by editing the translated version to match the original English version. However, the translation was not checked nor authorized by the authors of the CBCL. Cross-cultural norms are available for the CBCL, which were applied to obtain the T-scores reported in our study. Additionally, the CBCL has proven sensitive to exposure and intervention effects of pediatric HIV in out other published work (Bangirana *et al*. 2009*a*, *b*; Boivin *et al*. 2013).

Caregivers were also interviewed with the BRIEF for school-aged children. This instrument is specifically designed to measure the range of executive function behaviors in children with 86-items in 8 non-overlapping clinical scales (Inhibit, Shift, Emotional Control, Initiate, Working Memory, Plan/Organize, Organization of Materials and Monitor). The BRIEF is divided into two broad indexes, each of which is derived from specific clinical scales; Metacognition Index (Monitor, Organization of Materials, Plan/Organize, Working Memory, Initiate) and Behavioral Regulation (Emotional Control, Shift, Inhibit). A Global Executive Composite score takes into account all clinical scales and represents the child's overall executive function. Publisher copyright permission was obtained for the BRIEF and was translated, as specified by the publisher (PAR, Inc.). The final version was approved by one of the test authors (Peter Isquith) after several iterations.

### Statistical analyses

Demographic characteristics and symptom endorsement frequencies were calculated. Internal consistency of the BRIEF and CBCL scales were evaluated using Cronbach**’**s α. Simple- (SLR) and multiple-linear regression (MLR) modeling was used to relate BRIEF and CBCL scores to demographic characteristics (sex and age), ART status, viral load and immunologic biomarkers (CD4^+^ cell count, CD4^+^ CD38^+^ HLADR^+^ and CD8^+^ CD38^+^ HLADR^+^). All statistics were two-sided. Models for MLR were constructed on the basis of statistical significance (*p* < 0.05) of the association between outcome and variable using SLR, and were added to the final model using the forward stepwise selection method. All analyses were performed in STATA version 12 [*Stata* (computer program). Version 12. College Station, TX 2012].

## Results

A total of 144 HIV infected children underwent neuropsychological assessment as part of the parent study. Table 1 presents the demographic characteristics of the study sample and summarizes their immunological parameters and neuropsychological test-scores. The mean age of the study sample was 8.9 years (s.d. = 1.9 years) and gender distribution was equal (50% male). Participants’ mean CD4^+^ CD38^+^ HLADR^+^ T-cell activation was 5.7% (s.d. = 5.1%), CD 8^+^ CD38^+^ HLADR^+^ T-cell activation was 17.5% (s.d. = 11.2%). CD4 cell % mean was 35.1% (s.d. = 15.0%), one child had a viral load of <50 copies/ml and 38 participants (26.4%) had plasma HIV RNA of >100 000 copies/ml (see Table 1).

**Table 1.** Demographic characteristics, immunological parameters and CBCL and BRIEF scores of school-age children living with HIV in Kayunga, Uganda

Characteristic	School-age children living with HIV *N* = 144 (%)
Age in years, mean (s.d.)	8.9 (1.9)
Gender	
Male	72 (50)
Female	72 (50)
On ART	
Yes	82 (56.9)
OI prophylaxis	
Yes	144 (100)
CD 4^+^ CD38^+^ HLADR^+^ , % mean (s.d.)	5.7 (5.1)
CD 8^+^ CD38^+^ HLADR^+^ %mean (s.d.)	17.5 (11.2)
CD4 T cell %, mean (s.d.)	35.1 (15.0)
Plasma HIV RNA copies/ml	
<4000	60 (41.7)
4001–20 000	18 (12.5)
20 001–55 000	12 (8.3)
55 001–100 000	16 (11.1)
>100 000	38 (26.4)
CD 4/CD 8 ratio (s.d.)	0.8 (0.5)
BRIEF	
Behavioral Regulation Index *T* score (s.d.)^a^	46.0 (8.5)
Metacognition Index *T* score (s.d.)^a^	48.8 (9.3)
Global Executive Composite Index *T* score (s.d.)^a^	47.9 (9.5)
CBCL	
Internalizing Problems *T* score (s.d.)^b^	60.9 (9.0)
Externalizing Problems *T* score (s.d.)^b^	58.9 (7.9)
Total Problems *T* score (s.d.)^b^	58.5 (8.5)

OI, opportunistic infections; ART, antiretroviral therapy.

a Behavior Rating Inventory of Executive Function (BRIEF) parent form for children 5–18 years. T scores (adjusted for age and gender using American norms).

b Achenbach Child Behavior Checklist (CBCL) parent form for children 6–18 years T scores (adjusted for age and gender using cross-cultural norms).

Table 1 also shows the BRIEF and CBCL scores. Mean Global Composite Index of the BRIEF was 46 (s.d. = 9.5), while the mean TP Scale score of the CBCL was 58.5 (s.d. = 8.5). Out of 144 participants, 7 (4.9%) in the Global Executive Composite Index and 36 (25%) in the TP Scale had a score ≥65, score that is associated with higher likelihood of executive dysfunction.

Internal reliability (Cronbach's α) was 0.88 for the BRIEF and 0.76 for the CBCL.

Results from linear regression analyses are presented in Tables 2 and 3. Regression models for BRIEF were adjusted for gender, age, ART status, CD4 T-cell activation and viral load. Higher behavioral regulation index scores on the BRIEF were associated with higher viral loads (aβ = 16.7 × 10^−6^, 95% CI −5.00 × 10^−6^ to 28.4 × 10^−6^) (Table 2). Children who were older had lower Metacognition Index (MI) scores (e.g. less capacity to plan, working memory and organize) when compared with younger ones (aβ = −1.06, 95%CI −2.02 to −0.09) (Table 2).

**Table 2.** Unadjusted and adjusted linear regression models for BRIEF among 144 school-age children living with HIV in Kayunga, Uganda

BRIEF				
Behavioral regulation index				
	Unadjusted		Adjusted	
Variable	β (95% CI)	*P* value	aβ (95% CI)	*p* value
Sex	1.82 (−1.50 to 5.15)	0.28	1.68 (−1.65 to 5.02)	0.32
Age	−0.21 (−1.1 to 0.68)	0.64	−0.22 (−1.12 to 0.68)	0.57
On ART	0.80 (−2.60 to 4.21)	0.64	1.77 (−1.71 to 5.25)	0.24
CD 4^+^ CD38^+^ HLADR^+^	0.04 (−0.32 to 0.40)	0.82	−0.32 (−0.75 to 0.11)	0.17
Viral load	**11.0 × 10^−6^ (1.73 × 10^−6^–20.3 × 10^−6^)**	**0.02**	**16.7 × 10^−6^ (5.0 × 10^−6^–28.4 × 10^−6^)**	**<0.01**
Metacognition index				
Sex	2.84 (−0.77 to 6.44)	0.12	3.16 (−0.43 to 6.75)	0.88
Age	−**1.12 (**−**2.07 to** −**0.16)**	**0.02**	−**1.06 (**−**2.02 to** −**0.09)**	**0.03**
On ART	1.78 (−1.91 to 5.49)	0.34	1.70 (−2.04 to 5.45)	0.37
CD 4^+^ CD38^+^ HLADR^+^	−0.01 (−0.39 to 0.38)	0.98	−0.17 (−0.63 to 0.30)	0.48
Viral load	4.99 × 10^−6^ (−5.34 × 10^−6^ to 15.3 × 10^−6^)	0.34	8.84 × 10^−6^ (−3.79 × 10^−6^–21.5 × 10^−6^)	0.17
Global executive composite index				
Sex	3.16 (−0.51 to 6.83)	0.09	3.34 (−0.34 to 7.03)	0.08
Age	−0.78 (− 1.76 to 0.21)	0.12	−0.72 (−1.72 to 0.27)	0.12
On ART	1.96 (−1.81 to 5.73)	1.02	2.40 (−1.44 to 6.24)	0.22
CD 4^+^ CD38^+^ HLADR^+^	0.03 (−0.37 to 0.42)	0.88	−0.23 (−0.70 to 0.24)	0.44
Viral load	7.87 × 10^−6^ (−2.60 × 10^−6^–18.3 × 10^−6^)	0.14	12.6 × 10^−7^ (−0.37 × 10^−6^–25.5 × 10^−6^)	0.06

ART, antiretroviral therapy

a T scores were used for simple and multiple linear regression models.

Statistically significant values appear in bold

**Table 3.** Unadjusted and adjusted linear regression models for CBCL among 144 school-age children living with HIV in Kayunga, Uganda

CBCL				
Internalizing problems				
	Unadjusted		Adjusted	
Variable	β (95% CI)	*p* value	aβ (95% CI)	*p* value
Sex	**4.75 (1.73–7.77)**	**<0.01**	**4.48 (1.42–7.54)**	**<0.01**
Age	−0.28 (−1.11 to 0.56)	0.52	−0.41 (−1.24 to 0.41)	0.32
On ART	−2.36 (−5.49 to 0.76)	0.14	−1.38 (−4.59 to 1.83)	0.40
CD 8^+^ CD38^+^ HLADR^+^	**0.16 (0.02–0.30)**	**0.03**	0.12 (−0.03 to 0.27)	0.11
Externalizing problems				
Sex	1.05 (−1.70 to 3.81)	0.45	0.46 (−2.34 to 3.27)	0.74
Age	−0.30 (−1.04 to 0.43)	0.42	−0.47 (−1.22 to 0.29)	0.22
On ART	−0.02 (−2.77 to 2.80)	0.99	1.01 (−1.93 to 3.96)	0.45
CD 8^+^ CD38^+^ HLADR^+^	**0.14 (0.02–0.27)**	**0.02**	**0.17 (0.04–0.31)**	**0.01**
Total problems				
Sex	**3.97 (1.09–6.84)**	**<0.01**	**3.65 (0.71–6.58)**	**<0.01**
Age	−0.46 (−1.25 to 0.32)	0.25	−0.55 (−1.33 to 0.24)	0.17
On ART	0.05 (−3.03 to 2.92)	0.98	0.89 (−2.18 to 3.96)	0.57
CD 8^+^ CD38^+^ HLADR^+^	**0.14 (0.01–0.27)**	**0.04**	0.15 (0.00–0.28)	0.05

ART, antiretroviral therapy

a T scores were used for simple and multiple linear regression models.

Statistically significant values appear in bold

Regression models for CBCL were adjusted for gender, age, ART status and CD8T-cell activation. Gender was associated with higher Internalizing and TPs scores (aβ = −4.48, 95%CI −1.42 to 7.54). Girls scored on average 4.48 points higher in the Internalizing scale and 3.65 points in the TPs scale (i.e. more behavior problems) (Table 3). Higher percentage of CD8^+^ CD38^+^ HLADR^+^ were associated with higher scores in the EPs Scale (aβ = −0.17, 95% CI −0.04 to 0.31) and the TPs Scale (aβ = −0.15, 95% CI −0.00 to 0.28) of the CBCL (Table 3).

## Discussion

This study showed that clinically stable children living with HIV in Uganda have more behavioral and emotional problems as reported by their parents in the BRIEF (executive function) and CBCL (psychiatric symptoms) questionnaires with increasing viral loads and higher percentages of CD8^+^ T-cells expressing CD38^+^ and HLADR^+^. To the best of our knowledge, this study represents one of the first to evaluate the extent to which the immunological features of HIV disease are predictive of behavioral and psychiatric symptoms in rural school-age children in a sub-Saharan African setting. These findings are comparable with studies in the USA and elsewhere demonstrating observable behavioral sequelae of HIV disease in young children (Mellins *et al*. 2003; Wolters & Brouwers, 2005).

The strongest correlates of increased behavioral symptoms in this study were CD8^+^ T-cells expressing CD38^+^ and HLADR^+^ and viral load, other factors were consistent with previous studies that have reported demographic characteristics, such as gender, to be strongly correlated with behavioral problems (Mellins *et al*. 2003).

We found that parents are more likely to report Internalizing Problems for girls than boys for the CBCL, which is consistent with what has been reported in most countries (Rescorla *et al*. 2007), but in disagreement with other studies reporting no gender differences in pre-school children (Keiley *et al*. 2000; Shala & Dhamo, 2013). Regarding age, we observed lower values with increasing age on the MI of the BRIEF values (i.e. lower levels of executive dysfunction), corresponding with the development of cognitive abilities such as memory, comprehension and learning processes taking place from pre-school to adolescence (Händel *et al*. 2013).

Although the pathogenic mechanism of CNS disease is not fully understood, immune activation seems to play a fundamental role, with CD8^+^ T-cells continually crossing the blood-brain barrier and promoting activation of infected cells, which in turn lead to a pro-inflammatory cascade, resulting in further damage (Kovacs, 2009). Research suggests that having higher CD8^+^ CD38^+^ HLADR^+^ cells is associated with having poorer neurobehavioral outcomes (McCloskey *et al*. 2001). Children with higher percentages of CD8^+^ T-cells expressing CD38^+^ and HLADR^+^ had more psychiatric symptoms (higher scores) in the EPs and in the TPs scale of the CBCL, suggesting poorer emotional and behavioral outcomes. We found no association between CD4^+^ CD38^+^ HLADR^+^ and the BRIEF or the CBCL.

More psychiatric symptoms in the EPs and TPs scales of the CBCL were detected in children who had a higher percentage of CD8^+^ CD38^+^ HLADR^+^. These findings are consistent with previous research reported by Mekmullica *et.al*. who found that the absence of immune activation as measured by having ≤5% CD8^+^ HLADR+ cells was associated with better performance on the Psychomotor Developmental Index of the Bayley scale through the third year of life (Mekmullica *et al*. 2009). However, this is inconsistent with research by Kapetanovic *et.al*. who suggested that CD8^+^ CD38^+^ HLADR^+^ cells had a neuroprotective effect among children <1 year of age (Kapetanovic *et al*. 2012). Our findings suggest that school aged children with higher percentage of CD8^+^ CD38^+^ HLADR^+^ cell activation are more likely to have a negative behavioral outcomes as measured by the CBCL which is consistent with the concept that CD38^+^ and HLADR^+^ are observed in advanced disease stages (McCloskey *et al*. 2001).

Viral load has been previously described as a factor that impacts a child's behavior. Jeremy *et.al*. reported poor neuropsychological functioning in HIV infected children with high viral loads (>50 000 copies/ml), specifically, having a high viral load was associated with difficulties on children's emotional control, task shifting and inhibition (Jeremy *et al*. 2005). Similarly, in our study, higher viral loads were associated with higher scores on the Behavior Regulation Scale of the BRIEF, encompassing difficulties with emotional control, task shifting and inhibition. Although the magnitude of this increment on the Behavior Regulation Scale was small, we think that this could have clinical significance that could guide preventive interventions and treatment services.

Further research is needed to elucidate the immunopathological pathways through which executive function, as collected on the BRIEF, can be affected by HIV, which could specifically be associated with HIV cognitive impairment. Longitudinal studies could contribute to our understanding on how ART and viral load fluctuations across time can affect behavior in children.

In contrast to previous findings, we did not find an association between CD4^+^ CD38^+^ HLADR^+^ and the BRIEF or CBCL. Kapetanovic *et al*. reported a positive association between both CD4^+^ CD38^+^ HLADR^+^ and CD4^+^ CD38^−^ HLADR^+^ cells and neurodevelopmental outcomes as measured with the full-scale IQ (FSIQ) from Bayley scales of infant development (BSID-II) and Wechsler test and a negative association on the same test with CD4^+^ CD38^+^ HLADR^−^ cells, suggesting that CD4^+^ cell activation status could play a role in the development of neurocognitive deficits (Kapetanovic *et al*. 2012).

### Limitations

Our results must be viewed in light of several limitations. First, results are based on cross-sectional data and therefore we cannot state causality of the observed associations. However, our results are supported by previous studies with similar findings (Jeremy *et al*. 2005; Mekmullica *et al*. 2009).

In our bivariate and multiple logistic regression models, we were unable to explore and adjust for sociodemographic (e.g. delinquent behavior, age, school competence, orphans) and economic factors (e.g. low income) that have been reported to impact on the association between HIV and behavioral and emotional factors as measured by the CBCL and BRIEF (Bomba *et al*. 2010; Tadesse *et al*. 2012; Mellins & Malee, 2013; Llorente *et al*. 2014).

Although standardized norms for these neuropsychological measures are not available for Uganda for the BRIEF, the standardized scores are only used for research purposes within the study sample to assess the correspondence between immunological factors and child measures internally.

Given that neurobehavioral assessment was the primary goal of the randomized control trial, limited markers of T-cell activation were collected, limiting our capacity to compare between different T-cell subsets or to include additional markers in the analysis. Although a wider range of T-cell activation markers would have provided additional information regarding the role that immune cell activation has on neurobehavioral outcomes of aging children living with HIV, we believe the results presented here add to the role that commonly used T-cell activation markers have as predictors of behavioral problems within this group.

Finally, this analysis was limited to the assessment of limited individual sociodemographic, clinical and immunological factors as correlates of neurobehavioral outcomes. Future research should aim to assess economic, environmental and social factors (Gadow *et al*. 2012) that could impact behavior among children living with HIV, as well as a detailed assessment of factors related to ART (e.g. time of initiation and adherence) that could also modify behavioral outcomes (Laughton *et al*. 2013; Naar-King *et al*. 2013).

## Conclusion

Identifying HIV-1 infected children who are at greatest risk of poorer behavioral outcomes is critical for timely and optimal therapeutic and preventive care. Viral load appears to be a plausible virological marker given that it is now being routinely collected in resource constrained settings.

Given that neurodevelopmental problems among children living with HIV are likely to persist as they move onto adolescence and adulthood, it is important to identify behavioral problems within this group and to provide access to comprehensive psychosocial care and support aiming to prevent risky sexual behavior, substance use and to promote ART adherence (Laughton *et al*. 2013; Mellins & Malee, 2013).
